# Targeting of vascular adhesion protein-1 by positron emission tomography visualizes sites of inflammation in *Borrelia burgdorferi*-infected mice

**DOI:** 10.1186/s13075-017-1460-4

**Published:** 2017-11-22

**Authors:** Riikka Siitonen, Annukka Pietikäinen, Heidi Liljenbäck, Meeri Käkelä, Mirva Söderström, Sirpa Jalkanen, Jukka Hytönen, Anne Roivainen

**Affiliations:** 1 Turku PET Centre, University of Turku, Kiinamyllynkatu 4-8, FI-20520 Turku, Finland; 2 Institute of Biomedicine, University of Turku, Kiinamyllynkatu 10, FI-20520 Turku, Finland; 3 Turku Doctoral Programme for Molecular Medicine, Turku, Finland; 4 Turku Center for Disease Modeling, University of Turku, Kiinamyllynkatu 10, FI-20520 Turku, Finland; 5 Department of Pathology, Turku University Hospital, Kiinamyllynkatu 10, FI-20520 Turku, Finland; 6 MediCity Research Laboratory, University of Turku, Tykistönkatu 6, FI-20520 Turku, Finland; 7 Microbiology and Genetics Department, Turku University Hospital, Kiinamyllynkatu 10, FI-20520 Turku, Finland; 8 Turku PET Centre, Turku University Hospital, Kiinamyllynkatu 4-8, FI-20520 Turku, Finland

**Keywords:** Lyme borreliosis, *Borrelia burgdorferi*, arthritis, PET, Infection, Inflammation, Siglec-9, VAP-1

## Abstract

**Background:**

In the present study, we sought to evaluate the feasibility of targeting vascular adhesion protein-1 (VAP-1) by positron emission tomography (PET) for the longitudinal quantitative assessment of *Borrelia burgdorferi* infection-induced inflammation in mice.

**Methods:**

Mice with *B. burgdorferi* infection-induced arthritis were studied. During a 7-week follow-up period, the progression of arthritis was monitored weekly with ^68^Ga-DOTA-Siglec-9 PET/computed tomography (CT) and measurement of tibiotarsal joint swellings. A subgroup of infected mice was treated with ceftriaxone. Finally, histopathological assessment of joint inflammation was performed and VAP-1 expression in joints were determined.

**Results:**

Explicit joint swelling and ^68^Ga-DOTA-Siglec-9 uptake could be demonstrated in the affected joints from *B. burgdorferi*-infected mice. By contrast, no obvious accumulation of ^68^Ga-DOTA-Siglec-9 was detected in joints of uninfected mice. The maximum swelling and highest uptake in the affected joints were observed 4 weeks after the infection. ^68^Ga-DOTA-Siglec-9 uptake in joints correlated with joint swelling (*P* < 0.0001) and histopathological scoring of inflammation (*P* = 0.020). Despite short-term antibiotic treatment, the arthritis persisted, and the PET signal remained as high as in nontreated mice. Immunohistochemistry revealed strong-to-moderate expression of VAP-1 in the synovium of *B. burgdorferi*-infected mice, while only weak expression of VAP-1 was detected in uninfected mice.

**Conclusions:**

The present study showed that ^68^Ga-DOTA-Siglec-9 can detect *B. burgdorferi* infection-induced arthritis in mice. Furthermore, longitudinal PET/CT imaging allowed monitoring of arthritis development over time.

## Background

Lyme borreliosis (LB) is a human tick-borne infection caused by the spirochete of *Borrelia burgdorferi* sensu lato (*B. burgdorferi*) [1]. Localized infection at the tick bite site, the so-called erythema migrans skin lesion, is the most common manifestation of LB. From the inoculation site in the skin, dissemination of bacteria throughout the body occurs via the blood circulation. In the early-disseminated stage of infection, manifestations can occur in the nervous system and heart. Late LB usually manifests as a chronic skin infection called acrodermatitis chronica atrophicans or as Lyme arthritis (LA), the *B. burgdorferi* infection of joints [2, 3]. Antibiotic treatment eradicates the bacteria and cures the infection in most patients, but some patients have symptoms that persist after the antibiotic treatment [4]. Currently, no in vivo imaging techniques allow the specific visualization of *B. burgdorferi* infection in humans.

C3H mice are widely used as an animal model to study the dissemination and treatment response of *Borrelia* infection. *B. burgdorferi*-infected C3H mice develop joint symptoms resembling those of human disease, including joint swelling, synovial hypertrophy and hyperplasia, and leukocyte infiltration [5]. Although there are a few in vivo imaging techniques that can be used to monitor *B. burgdorferi* infection in mice, these techniques have some disadvantages. Fluorescence- or bioluminescence-based imaging techniques may visualize the infection at the level of the whole animal or even at a particular tissue, but they require genetically engineered *B. burgdorferi* that express green fluorescent protein or luciferase [6–9]. Offering an alternative to current in vivo imaging techniques, positron emission tomography (PET) allows longitudinal imaging of individual mice at several time points. This presents the opportunity to visualize and quantify the status of specific organs; monitor the dissemination of an infection; and use wild-type and unlabeled bacteria, which is especially important.

The gold standard for PET imaging of infectious and inflammatory diseases is the ^18^F-labeled glucose analog fluorodeoxyglucose (^18^F-FDG). The accumulation of ^18^F-FDG is dependent on the glycolytic activity of inflammatory cells because these cells use glucose as an energy source during activity. In addition, increased glucose accumulation in inflamed tissues occurs in relation to the high numbers of glucose transporters on the cell membranes of inflammatory cells [10]. However, ^18^F-FDG is not an inflammation-specific tracer, because it accumulates in any cells that use glucose as an energy source. Over the years, attempts have been made to develop more specific radiopharmaceuticals for imaging of inflammation and infection, but these have generally met with little success [11–13].

Leukocyte migration from the blood to tissue is a crucial step in acute and chronic inflammation. Vascular adhesion protein-1 (VAP-1) is an endothelial cell molecule that participates in leukocyte recruitment in inflamed tissue. Under normal conditions, VAP-1 is stored in intracellular storage granules in endothelial cells, but upon inflammation, it is rapidly translocated from the intracellular storage granules to the endothelial cell surface [14]. However, though VAP-1 has an important role during early inflammation, its expression on the endothelial surface remains constant for a longer time if inflammation continues. Besides being an adhesion molecule, VAP-1 (currently also known as amine oxidase, copper containing 3, or AOC3) has enzymatic activity. The end products of the VAP-1-catalyzed enzymatic reaction are inflammation mediators [15]. Therefore, this makes VAP-1 a potential target for anti-inflammatory therapy and in vivo imaging of inflammation. We have published several articles concerning VAP-1 as a target for in vivo imaging in different experimental animal models [16–21]. Although leukocytes can bind to the endothelium in a VAP-1-dependent manner, counterreceptors of VAP-1 were for a long time unknown. A few years ago, it was discovered that the sialic acid-binding immunoglobulin-like lectins (Siglecs) 9 and 10 are leukocyte ligands of VAP-1 [22, 23]. Previously, we demonstrated that VAP-1-targeted gallium-68-labeled Siglec-9 motif containing peptide (^68^Ga-DOTA-Siglec-9) can be used for PET imaging of inflammation and cancer [22, 24, 25].

Expression levels of the adhesion molecules P-selectin, intercellular adhesion molecule-1, and vascular cell adhesion molecule-1 are elevated in the synovia of *B. burgdorferi*-infected mice [26, 27]. A marked similarity is noted in the expression of these adhesion molecules between human LA and the mouse model of LA. In addition, VAP-1 is highly expressed in the synovia of patients with treatment-resistant LA, suggesting that a VAP-1-targeting tracer such as ^68^Ga-DOTA-Siglec-9 could be useful in the imaging of LA [28].

The purposes of this study were (1) to evaluate the feasibility of VAP-1-targeting ^68^Ga-DOTA-Siglec-9 for the longitudinal quantitative monitoring of *B. burgdorferi* infection-induced joint inflammation in mice and (2) to compare the results obtained using this tracer with the traditional indicators of arthritis in mice, including joint swelling and histological scoring of joint inflammation. Furthermore, we assessed whether the tracer could detect ceftriaxone treatment-induced changes in inflammation.

## Methods

### *B. burgdorferi* strains

A wild-type *B. burgdorferi* sensu stricto N40 strain, which was provided by Sven Bergström, University of Umeå, Sweden, was used to infect mice. The spirochetes were cultivated in Barbour-Stoenner-Kelly II (BSK II) medium at 33 °C.

### Animal model and experimental design

All animal experiments were approved by the national animal experiment board in Finland and the Regional State Administrative Agency for Southern Finland, and they were conducted in accordance with the European Union directive. To induce LB in mice, 22 four-week-old female C3H/HeNhsd mice (Envigo, Horst, The Netherlands) were infected with 10^6^ bacteria in 100 μl of PBS via an intracutaneous injection into the lower back. Four C3H/HeNhsd mice were given injections with 100 μl of PBS into the lower back to act as uninfected controls. Eight of the *B. burgdorferi*-infected mice were treated with ceftriaxone (Rocephalin®; Roche, Grenzach-Wyhlen, Germany) starting at 4 weeks after infection. A dose of 25 mg/kg ceftriaxone was subcutaneously administered twice daily for 5 days. The swelling of hind tibiotarsal joints (mediolateral diameter) was monitored weekly using a metric caliper. The measurer was blinded to the identity of the groups.

Six mice (two *B. burgdorferi*-infected, two uninfected controls, and two ceftriaxone-treated infected mice) were imaged with PET/computed tomography (CT) each week, starting at 4 days postinfection. After the last PET/CT imaging at week 7, the mice were killed, and tissues were collected for ex vivo gamma counting, histology, and anti-VAP-1 immunohistochemistry. In addition, starting at week 2, two *B. burgdorferi*-infected and/or two ceftriaxone-treated infected mice were killed after longitudinal PET/CT imaging each week for ex vivo gamma counting of tracer uptake in several excised organs. After gamma counting, ear, heart, hind tibiotarsal joint, and urinary bladder samples were subjected to *Borrelia* culture, and one tibiotarsal joint sample was collected for histological analysis.

### *B. burgdorferi* culturing of tissue samples

Ear, heart, tibiotarsal joint, and urinary bladder samples were collected to assess the *B. burgdorferi* infection status. All instruments were disinfected in ethanol between dissections of different samples. The tissue samples were cultured in BSK II medium supplemented with phosphomycin (50 μg/ml; Sigma-Aldrich, Steinheim, Germany) and rifampicin (100 μg/ml; Sigma-Aldrich) at 33 °C for 6 weeks. Growth was monitored every 2 weeks with a dark-field microscope.

### Histology and scoring

One tibiotarsal joint of each mouse was formalin-fixed, demineralized, paraffin-embedded, and cut into 5-μm sections for hematoxylin and eosin staining. Joint inflammation was analyzed by an experienced pathologist blinded to the infection status of the mice. Synovial proliferation, chronic inflammation, and active inflammation were scored on a scale from 0 to 6. The diameters of joints were also measured.

### Immunohistochemistry

Formalin-fixed, paraffin-embedded, decalcified joints were stained with a monoclonal TK10-79 antibody recognizing mouse VAP-1 or a negative class-matched control antibody (both 1 μg/ml). Antigen retrieval with 0.01 M citric acid (pH 6.0) and removal of endogenous peroxidase with 1% H_2_O_2_ were performed. The primary antibody was detected with a VECTASTAIN Elite ABC rat immunoglobulin G kit (PK-6104; Vector Laboratories, Burlingame, CA, USA), followed by horseradish peroxidase-conjugated avidin. Diaminobenzidine (K3468; DAKO, Glostrup, Denmark) was used as a chromogen. The samples were counterstained with hematoxylin.

### Radiochemistry

A cyclic peptide, CARLSLSWRGLTLCPSK, with disulfide-bridged cysteines consisting of residues 283–297 from the Siglec-9 and 8-amino-3,6-diooxaoctanoyl linker (polyethylene glycol derivative) between DOTA chelator and peptide (Peptide Specialty Laboratories GmbH, Heidelberg, Germany) was labeled with ^68^Ga as previously described [18, 22]. The radiochemical purity of ^68^Ga-DOTA-Siglec-9 was ≥ 95% throughout the study, as determined by reversed-phase radio-HPLC.

### PET/CT studies

Mice were fasted for 4 h with ad libitum access to water before PET/CT imaging. The mice were anesthetized with isoflurane; the tail vein was cannulated; and the urinary bladder was catheterized. The urinary bladder catheter was removed a few minutes before CT imaging. CT was performed for anatomical reference and attenuation correction. The mice were then subjected to intravenous injection with ^68^Ga-DOTA-Siglec-9 (9.9 ± 1.1 MBq) via the tail vein. Dynamic PET imaging was then performed for 30 minutes (Inveon Multimodality PET/CT System; Siemens Medical Solutions, Knoxville, TN, USA) starting from the time of injection. The PET data were acquired in a list mode and iteratively reconstructed with an ordered subset expectation maximization 3D algorithm, followed by maximum a posteriori reconstruction.

With CT used as the anatomical reference, quantitative PET analysis was performed by defining ROIs in hind tibiotarsal joints and other main organs using Inveon Research Workplace 4.1 software (Siemens Medical Solutions, Malvern, PA, USA). Two hind tibiotarsal joints from each mouse were analyzed individually at different time points. The uptake of ^68^Ga-DOTA-Siglec-9 was reported as the standardized uptake value (SUV), which was calculated as the average radioactivity concentration of the ROI corrected for the injected radioactivity dose and animal weight.

Immediately after the last ^68^Ga-DOTA-Siglec-9 PET/CT imaging, the mice were killed, and various tissues were excised and weighted. The radioactivity concentration of the tissues was measured with a gamma counter (Triathler3″; Hidex, Turku, Finland). The ex vivo radioactivity measurements were corrected for radionuclide decay from the time of injection and the weight of tissue and animal, and the results were expressed as percentage of injected radioactivity dose per gram of tissue.

### Statistical analysis

All data are expressed as mean ± SD. The normal distribution of the data was verified using the Shapiro-Wilk test. Student’s *t* test was used for normally distributed data. The Mann-Whitney *U* test was used for all other experiments. Comparisons between multiple groups were made using one-way analysis of variance with Dunnett’s correction. Pearson’s correlation coefficient was calculated for the association between two continuous variables. Nonparametric Spearman’s correlation was used to analyze associations between in vivo radioactivity and histological data. Statistical analyses were conducted using IBM SPSS Statistics version 23 software (IBM, Armonk, NY, USA).

## Results

### Characterization of *B. burgdorferi*-induced arthritis in mice

**Fig. 1:**
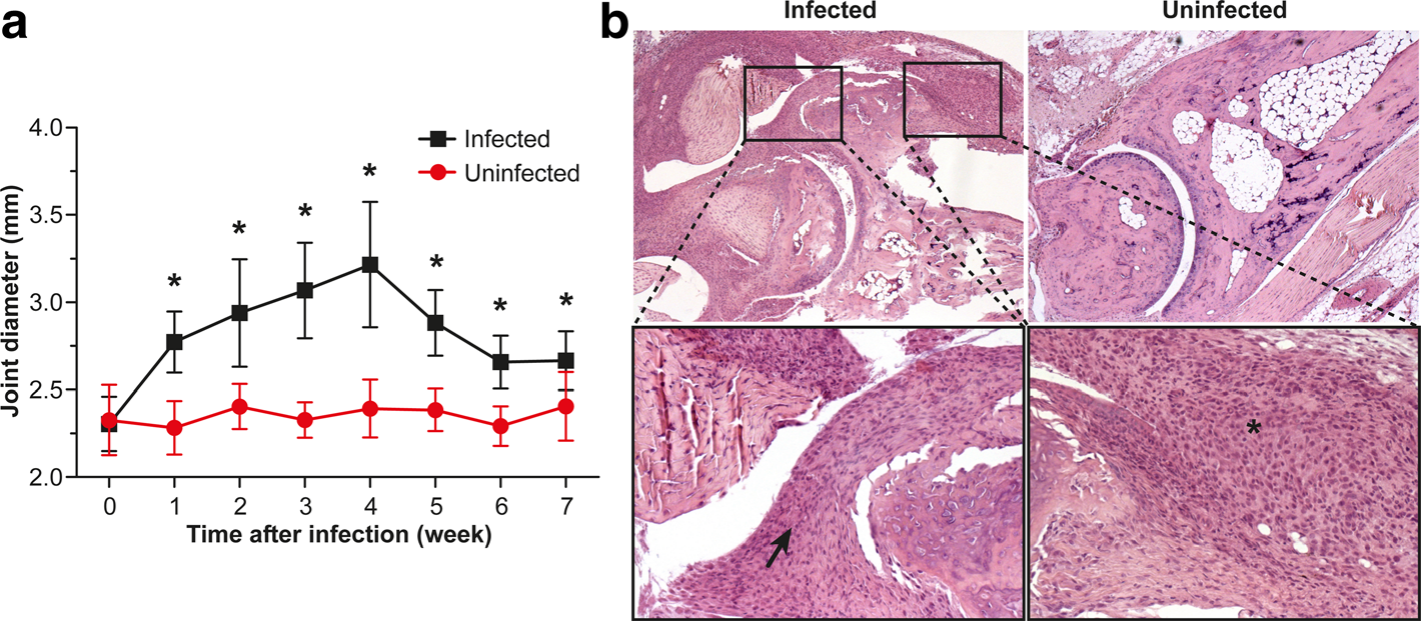
*Borrelia burgdorferi* causes severe arthritis. **a** Development of hind tibiotarsal joint swelling was monitored once a week by measuring the mediolateral diameter of the joint. The results from *B. burgdorferi*-infected mice are compared with those from uninfected mice. Values are mean ± SD; number of analyzed joints: infected (*n* = 8–28), uninfected (*n* = 8). *P < 0.05. **b** Representative images of tibiotarsal joints from a *B. burgdorferi*-infected (upper left) and an uninfected control (upper right). Lower insets show the indicated areas of infected mouse at higher magnification. Severe arthritis is seen in the infected mouse, with synovial proliferation (*arrow* in lower left image) and infiltration of inflammatory cells (asterisk in lower right image). Hematoxylin and eosin staining of paraffin-embedded 5-μm synovial tissue sections at × 50 and × 100 original magnification

The diameters of the tibiotarsal joints of all mice were measured at various time points throughout the experiment. The *B. burgdorferi-*infected mice developed visually explicit tibiotarsal joint swelling and redness. The maximum swelling was reached 4 weeks after the infection, with the swelling starting to decline after this point (Fig. 1a). The joint diameters of uninfected mice remained the same throughout the follow-up period.

All ear, heart, tibiotarsal joint, and urinary bladder samples of infected mice were *B. burgdorferi* culture-positive. These results verified that all infected mice had developed a disseminated *B. burgdorferi* infection. Tissue samples from uninfected control mice were culture-negative.

The histological findings from joints of infected mice revealed thickening of the synovial membrane, proliferation of synovial lining cells, and chronic inflammation that contained mainly lymphocytes (Fig. 1b). The scores of the tibiotarsal joints of infected mice reached a maximum at 5 weeks postinfection. Furthermore, synovial proliferation and chronic inflammation were also visible at the last time point (i.e., 7 weeks after infection). The tibiotarsal joints of uninfected mice were all scored as zero at week 7.

### Monitoring *B. burgdorferi* infection-induced inflammation in mice with longitudinal ^68^Ga-DOTA-Siglec-9 PET/CT

**Fig. 2:**
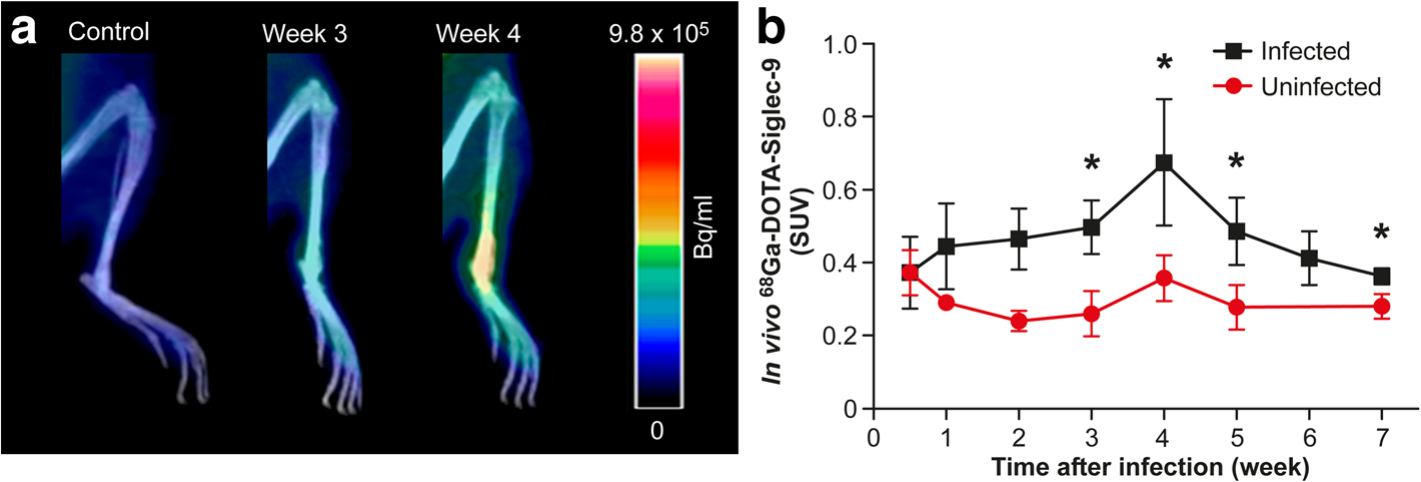
Uptake of gallium-68-labeled Siglec-9 motif containing peptide (^68^Ga-DOTA-Siglec-9) in mouse tibiotarsal joints. **a** Representative ^68^Ga-DOTA-Siglec-9 positron emission tomographic/computed tomographic images of an uninfected mouse and a *Borrelia burgdorferi*-infected mouse at weeks 3 and 4. **b** Quantification of in vivo tracer uptake in tibiotarsal joints of the infected and uninfected mice. Number of analyzed joints: infected (*n* = 4–8), uninfected (*n* = 4). *P < 0.05. *SUV* Standardized uptake value

^68^Ga-DOTA-Siglec-9 PET/CT was able to visualize *B. burgdorferi*-induced inflammation in the joints of infected mice, whereas uninfected mice showed only low accumulations of the tracer (Fig. 2a). Longitudinal assessment of the mice revealed that, in comparison with uninfected mice, the tracer signal in tibiotarsal joints of infected mice had already started to increase 1 week after the induction of infection. The highest tracer uptake was observed at 4 weeks postinfection (Fig. 2b). During the 3–5 weeks after the infection, the difference in uptake between the infected and uninfected mice was statistically significant (week 3 SUV, 0.50 ± 0.072 vs. 0.26 ± 0.063, *P* = 0.0040; week 4 SUV, 0.68 ± 0.17 vs. 0.36 ± 0.065, *P* = 0.0037; week 5 SUV, 0.49 ± 0.092 vs. 0.28 ± 0.063, *P* = 0.0067). The tracer uptake in the joints of infected mice peaked at week 4 and then started to decrease, but it remained higher than that in uninfected mice throughout the 7-week follow-up period (week 7 SUV, 0.36 ± 0.022 vs. 0.28 ± 0.034, *P* = 0.014). We did not see any differences in uptake in other organs, such as heart or brain, between the infected and uninfected mice (data not shown). In all studied mice, the excess of ^68^Ga-DOTA-Siglec-9 was eliminated rapidly via kidneys to the urinary bladder, which is in line with results of our previous studies [22, 24, 29].

The ex vivo gamma counting measurements of the excised tissue samples performed 30 minutes after tracer injection verified the in vivo PET/CT imaging results. Detailed results of the ^68^Ga-DOTA-Siglec-9 distribution in the tibiotarsal joints are shown in Table 1.

**Table 1:** ^68^Ga-DOTA-Siglec-9 uptake in mouse tibiotarsal joints detected using ex vivo gamma counting

	Tibiotarsal joints		
Weeks after infection	Infected	Ceftriaxone-treated infected	Uninfected
2	1.3 ± 0.13		
3	1.2 ± 0.26		
4	1.6 ± 0.24		
5	1.7 ± 0.27^a^	1.8 ± 0.25^a^	
6	0.96 ± 0.11^a,b^	1.4 ± 0.093	
7	1.6 ± 0.23^a,b^	1.1 ± 0.17	1.2 ± 0.067

The results are expressed as the percentage of injected radioactivity dose per gram of tissue (mean ± SD) measured at 30 minutes after injection of gallium-68-labeled Siglec-9 motif containing peptide (^68^Ga-DOTA-Siglec-9)

^a^ Significant difference in comparison with uninfected mice

^b^ Significant difference in comparison with ceftriaxone-treated infected mice

### Effect of ceftriaxone treatment at week 4 on joint inflammation

**Fig. 3:**
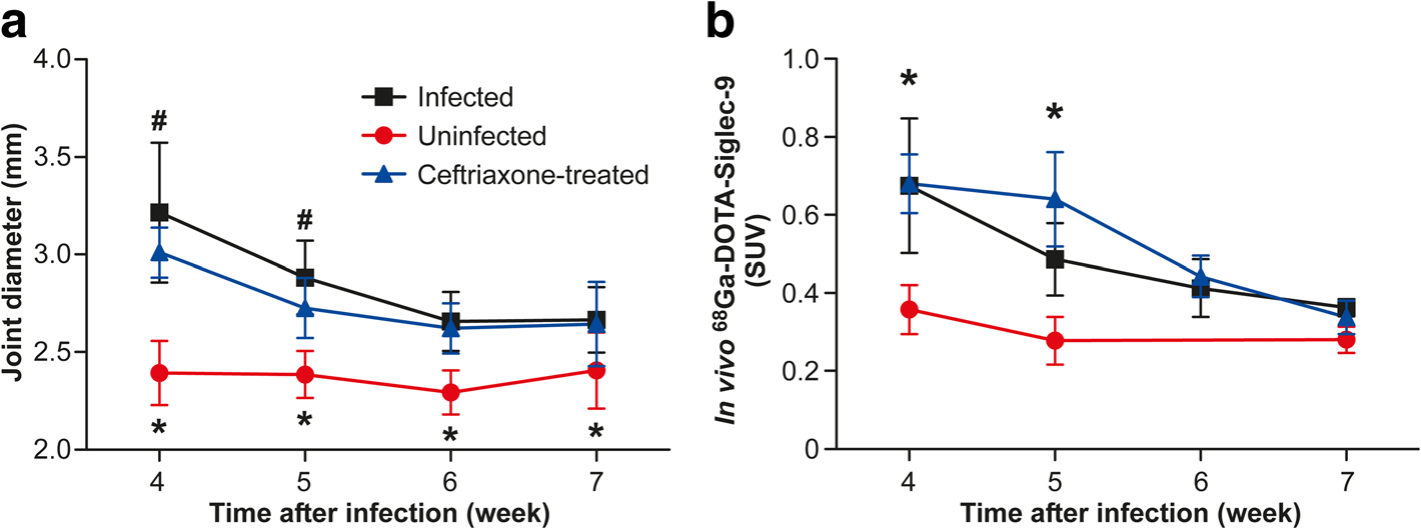
Evaluation of the tibiotarsal joints of ceftriaxone-treated mice. **a** Tibiotarsal joint swelling in the ceftriaxone-treated mice compared with the untreated infected and uninfected mice. Number of analyzed joints: untreated infected (*n* = 8–20), uninfected (*n* = 8), and ceftriaxone-treated (*n* = 8–16). **b** In vivo uptake values of Gallium-68-labeled Siglec-9 motif containing peptide (^68^Ga-DOTA-Siglec-9) in the ceftriaxone-treated mice compared with the untreated infected and uninfected mice. Number of analyzed joints: untreated infected (*n* = 4–8), uninfected (*n* = 4), and ceftriaxone-treated (*n* = 4–8). *P < 0.05 for ceftriaxone-treated vs. uninfected, ^#^ *P* < 0.05 for ceftriaxone-treated vs. infected. *SUV* Standardized uptake value

During the fourth week postinjection, a subset of *B. burgdorferi-*infected mice was treated with ceftriaxone twice daily for 5 days. Whereas *B. burgdorferi* were detected by culture from the ear, heart, tibiotarsal joint, and urinary bladder samples of untreated infected mice, samples of ceftriaxone-treated mice were culture-negative. The joint diameter of the ceftriaxone-treated mice started to decline simultaneously with the initiation of treatment, but the difference between the ceftriaxone-treated and uninfected mice remained statistically significant over the whole follow-up period (Fig. 3a). Furthermore, the differences in joint diameter between the ceftriaxone-treated and untreated infected mice were significant at weeks 4 and 5, but not after week 5, when the decline in both sets of mice was similar.

The ^68^Ga-DOTA-Siglec-9 uptake in tibiotarsal joints of the ceftriaxone-treated mice was in line with the swelling results. According to the in vivo PET imaging, the uptake of ^68^Ga-DOTA-Siglec-9 in tibiotarsal joints at weeks 4 and 5 was significantly higher in the ceftriaxone-treated mice than in the uninfected mice (Fig. 3b). The tracer uptake in tibiotarsal joints of the ceftriaxone-treated mice did not differ from that of the untreated infected mice. The ^68^Ga-DOTA-Siglec-9 uptake in the ceftriaxone-treated mice started to decline during the last weeks of the follow-up period (as was the case in the untreated infected mice), and it reached almost the same level as in the uninfected mice. The ex vivo results from tibiotarsal joints showed that the uptake was significantly higher in the ceftriaxone-treated mice than in the uninfected mice at week 5 (*P* = 0.0085) (Table 1). The uptake in tibiotarsal joints was also higher at week 6, although the difference did not quite reach statistical significance (*P* = 0.055).

### Histopathological evaluation correlates with in vivo ^68^Ga-DOTA-Siglec-9 PET

**Fig. 4:**
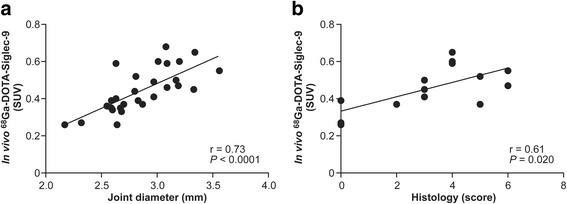
Correlations between joint swelling, histopathological changes, and positron emission tomography tracer uptake in mouse joints. Accumulation of gallium-68-labeled Siglec-9 motif containing peptide (^68^Ga-DOTA-Siglec-9) in the tibiotarsal joints in comparison with the joint diameter (**a**) and histology scores (**b**). Number of analyzed joints: infected (*n* = 24), uninfected (*n* = 4). Pearson’s and Spearman’s correlation coefficients and *P* values are presented. *SUV* Standardized uptake value

To determine whether the uptake of ^68^Ga-DOTA-Siglec-9 in the tibiotarsal joints correlated with histopathological changes in arthritis associated with *B. burgdorferi* infection, we performed correlation analyses between the individual in vivo PET results and swelling and histological scoring data. The individual ^68^Ga-DOTA-Siglec-9 uptake of the tibiotarsal joints correlated significantly with the joint diameters (*r* = 0.73, *P* < 0.0001) (Fig. 4a). Furthermore, the individual results of the histological scoring also showed a positive correlation with the in vivo PET results (*r* = 0.61, *P* = 0.020) (Fig. 4b).

### Expression of VAP-1 in mouse synovial tissue

**Fig. 5:**
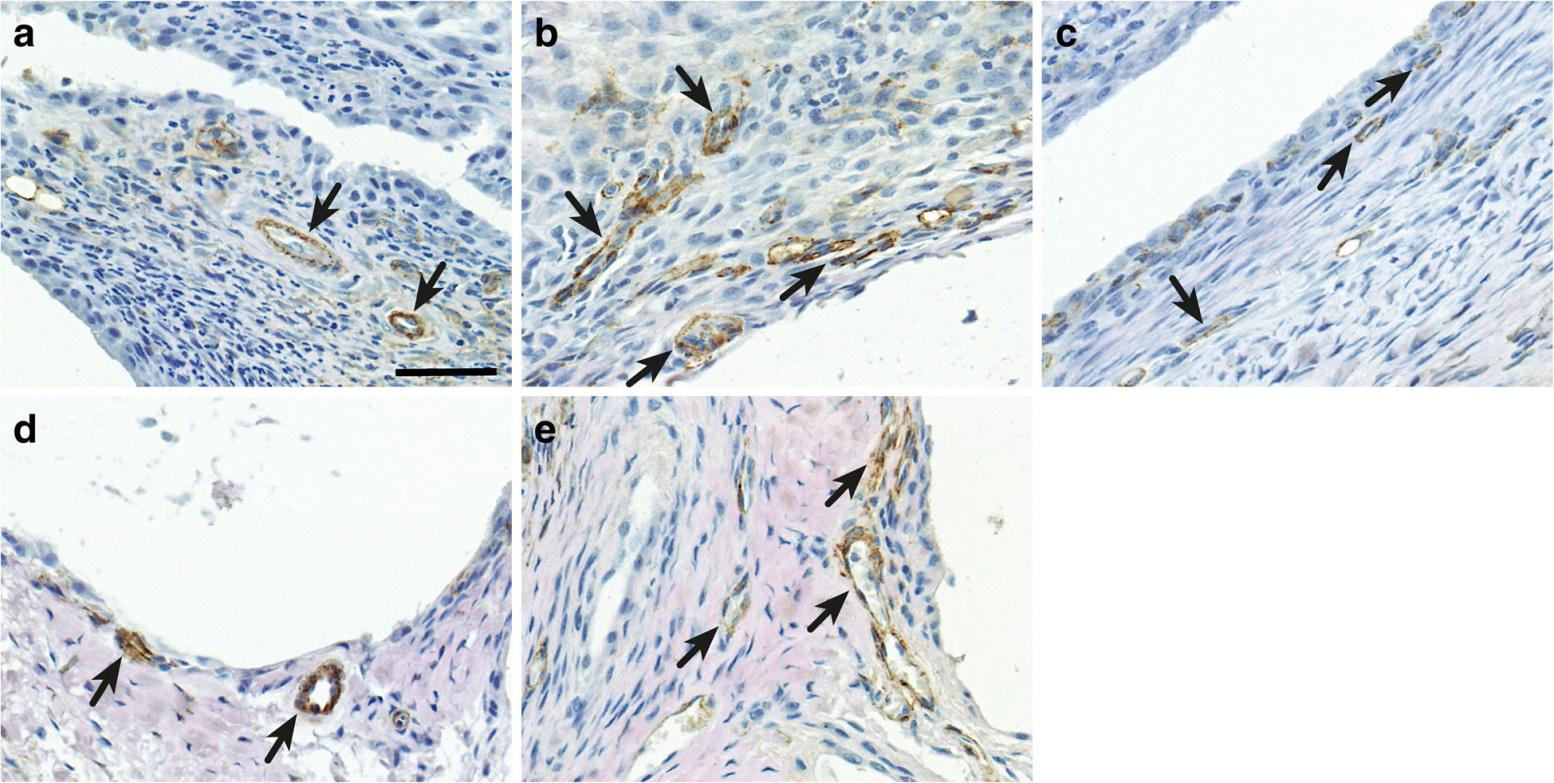
Expression of vascular adhesion protein-1 (VAP-1) in synovial tissue samples of mouse tibiotarsal joints. Representative VAP-1 immunohistochemical staining shows moderate staining of blood vessels at 2 weeks postinfection (**a**), more prominent staining at 4 weeks postinfection (**b**), and weak staining at 7 weeks postinfection (**c**). In the samples from the uninfected mice, only occasional VAP-1-positive blood vessels were observed (**d**). Immediately after the ceftriaxone treatment, expression of VAP-1 did not decline, but stayed at a moderate level (**e**). *Arrows* indicate VAP-1-positive blood vessels. Scale bar = 50 μm

To verify the expression of the tracer target in synovial tissue samples, longitudinal sections of tibiotarsal joints from infected, uninfected, and ceftriaxone-treated mice were immunostained for VAP-1. Moderate expression of VAP-1 in the synovial tissue vessels was detected in the samples from infected mice at 2 weeks postinfection (Fig. 5a). The strongest positive VAP-1 staining in synovial tissue was observed at 4 weeks postinfection (Fig. 5b), which was in line with the joint swelling and in vivo ^68^Ga-DOTA-Siglec uptake. The weak expression of VAP-1 in the synovial tissue vessels was observed at 7 weeks postinfection (Fig. 5c). Only occasional VAP-1-positive vessels were detected in the synovial tissue of the uninfected mice (Fig. 5d). After ceftriaxone treatment, the synovium showed moderate expression of VAP-1 (Fig. 5e). Thus, VAP-1 expression was evident in the synovial tissue of affected tibiotarsal joints.

## Discussion

In the present study, we focused on longitudinal quantitative PET/CT imaging with the VAP-1-targeting tracer ^68^Ga-DOTA-Siglec-9 to assess *B. burgdorferi* infection-induced inflammation in mice, with particular emphasis on the arthritic joints. This Siglec-9-derived cyclic peptide binds to VAP-1 [22]. Our results revealed that ^68^Ga-DOTA-Siglec-9 PET detected inflamed joints at an early stage and was able to monitor the fluctuating nature of *B. burgdorferi*-induced arthritis. By contrast, no obvious accumulation of ^68^Ga-DOTA-Siglec-9 was detected in the joints of uninfected mice. These findings were confirmed by the results of the ex vivo analyses, histological evaluations, immunohistochemical VAP-1 staining, and joint swelling measurements. Moreover, we showed that ^68^Ga-DOTA-Siglec-9 PET/CT imaging is also a suitable tool for the monitoring of treatment responses in mouse models of LB. Importantly, our results suggest that VAP-1-targeting PET has potential as a tool for imaging human patients with LA.

Mice are natural hosts for *B. burgdorferi* spirochetes and normally show no physiological signs of infection [1]. To study LB in vivo, mouse strains susceptible to infection by *B. burgdorferi* have been developed. The most commonly used mouse strain is C3H, which develops visually explicit joint symptoms with swelling and leukocyte infiltration [5]. In the mouse model of LB, the animals are usually infected with *B. burgdorferi* using 10^4^ to 10^7^ bacteria, which in most cases leads to prominent joint manifestations [5, 8, 30, 31]. In this study, C3H/HeNhsd mice were infected with 10^6^ bacteria because we wanted to ensure that the mice developed a disseminated infection with clear joint swelling. No other infectious doses were therefore evaluated.

In the present study, the peaks in ^68^Ga-DOTA-Siglec-9 uptake and joint swelling occurred at 4 weeks postinfection, with the uptake and inflammation then resolving over the next few weeks. It should be noted that the joint swelling of *B. burgdorferi*-infected mice rose sligthly at 7 weeks postinfection, which is in line with the results of our previous study [32]. Others have also reported that joint swelling and inflammation in mice peak 3–4 weeks postinfection [30, 32]. However, diameter measurements of tibiotarsal joints provide only limited information on the biological processes occurring in the joint. Conversely, molecular PET/CT imaging is a sensitive method and provides quantitative information on ongoing inflammatory processes and the status of the infection. In contrast to histopathological evaluation, the limited spatial resolution of PET/CT imaging cannot pinpoint the exact tissue and cellular level of the inflammatory process, but its high sensitivity enables the detection of inflammation at a very early stage.

The development of novel, noninvasive molecular imaging tools for the diagnosis and monitoring of infectious and inflammatory diseases is important. Clinically approved radiopharmaceuticals for the detection of infection are based on the influx of leukocytes during inflammation. The most commonly used PET tracer, the glucose analog ^18^F-FDG, has been used successfully in the detection of benign and malignant tumors as well as inflammatory and infectious conditions [33]. There are a few studies describing the use of ^18^F-FDG PET in brain manifestations of patients with LB infection [34–37], but whether the method is useful in diagnosing LB remains to be determined. Recently, we showed that ^18^F-FDG can be used to study *B. burgdorferi* infection-induced arthritis in mice [38]. Generally, ^18^F-FDG tends to give false-positive results, and the physiological uptake may mask the detection of inflammatory foci close to tissues that have high glucose metabolic activity. ^67/68^Ga-citrate was one of the earliest radiotracers to be made available for the imaging of infection, but the mechanism of accumulation at the site of infection is not entirely understood [39]. Autologous white blood cells labeled with long-lived radionuclides enable the detection of infectious processes or inflammatory foci [40] and are currently considered to be a leading imaging technique for targeting infection. Another interesting group of infection-selective radiotracers is the labeled siderophores, which have shown promising results for the imaging of infection caused by *Aspergillus fumigatus* [41]. Previous studies on imaging of *Mycobacterium tuberculosis* infections have also identified potential radiopharmaceuticals that may replace ^18^F-FDG in the future [42, 43]. Nevertheless, investigations on infection- and inflammation-selective radiopharmaceuticals are still in their infancy, and novel approaches need to be evaluated in different model systems.

Many radiopharmaceuticals have been investigated for the imaging of arthritic synovitis. The most promising results for the imaging of rheumatoid arthritis (RA) have been shown for stroma-targeting tracers, including methyl-^11^C-choline for targeting cell proliferation [44] and ^11^C-(*R*)-PK11195 for targeting 18 kDa translocator protein on activated macrophages [45]. In addition, several PET tracers have been explored for the imaging of RA in animal models, but their applicability to human RA has not been confirmed in human studies [46]. In the present study, we have shown that in vivo joint uptake of ^68^Ga-DOTA-Siglec-9 correlated well with histopathological scoring and joint diameter. These significant correlations with both parameters demonstrate that PET/CT is a suitable method for the in vivo imaging of infection-induced arthritis in mice. In addition, we can speculate that ^68^Ga-DOTA-Siglec-9 PET would be helpful for diagnosing LA in humans because VAP-1 is expressed in the synovium of patients with treatment-resistant LA [28].

The currently available techniques for studying LB, such as bioluminescence imaging, require animals to be killed at different time points for analyses of specific organs; this in turn increases the number of study animals required. PET imaging allows the same animal to be studied at multiple time points during the progression of disease and/or during therapy, which significantly reduces the number of animals required for a study. Furthermore, longitudinal intra-animal analyses can increase the statistical power of studies because, for example, both hind tibiotarsal joints can be analyzed simultaneously at different time points without variation due to the dissection of different animals. In vivo imaging may also improve the reliability of drug intervention studies because each animal or patient can be used as its own control by studying the subject before and after treatment.

Antibiotic treatments given in early LB usually eradicate the spirochetes and prevent late-stage manifestations in human patients. Ceftriaxone is the preferred drug for disseminated LB, and it is also effective in LA [1]. Oral or intravenous antibiotic treatment works well in patients with LA, and in most cases arthritis is healing. Previous studies using a mouse model of *B. burgdorferi* infection-induced arthritis indicated that ceftriaxone treatment eliminates live spirochetes and decreases joint swelling [31, 47]. However, the correct timing of treatment is essential if it is to have an effect on joint manifestations. When started at an early stage of infection (at 2 weeks), ceftriaxone treatment prevents the development of joint swelling [32]. During the later stages of infection, the timing of the treatment becomes a crucial factor with respect to its specific effects on joint manifestations. This is due to the fluctuating nature of *B. burgdorferi* infection-induced joint inflammation in mice. The joint swelling usually peaks a few weeks after the infection, with the inflammation then starting to spontaneously recover with normalization of the joint diameter. In the present study, we were unable to investigate the effect of ceftriaxone treatment with regard to joint inflammation. The treatment was initiated at 4 weeks postinfection, the time when a spontaneous decrease in joint swelling occurred in the untreated mice. However, a small increase in the in vivo uptake of ^68^Ga-DOTA-Siglec-9 in ceftriaxone-treated mice was observed at week 5 in comparison with the untreated mice. This was possibly induced by the debris of the dying spirochetes.

The observed differences of in vivo SUVs between the groups were small but still significant. This is based on the fact that PET can detect early biological changes with very high sensitivity [48]. In the present study, the performance PET/CT imaging in visualizing the effect of ceftriaxone treatment on mouse LA could not be investigated, owing to the timing of the treatment. Thus, the feasibility of ^68^Ga-DOTA-Siglec-9 PET/CT for detecting therapy response remains to be further investigated, such as with an early-stage treatment. Taken together, our findings support the concept of using VAP-1-targeting tracer for the imaging of synovial inflammation in LA, but further head-to-head studies are needed to confirm the advantages over ^18^F-FDG PET.

## Conclusions

We have demonstrated that ^68^Ga-DOTA-Sigec-9 can accurately detect *B. burgdorferi* infection-induced arthritis in mice. Longitudinal quantitative PET/CT imaging allows the monitoring of the development of arthritis over time. The present study shows the potential of the VAP-1-targeting imaging concept for identifying synovial inflammation and lays the foundation for further evaluation of the method in human patients with LA.

## Abbreviations

AOC3: Amine oxidase, copper containing 3
BSK II: Barbour-Stoenner-Kelly II medium
CT: Computed tomography
^18^F-FDG: ^18^F-labeled glucose analog fluorodeoxyglucose
^68^Ga-DOTA-Siglec-9: Gallium-68-labeled Siglec-9 motif containing peptide
LA: Lyme arthritis
LB: Lyme borreliosis
PET: Positron emission tomography
RA: Rheumatoid arthritis
Siglec: Sialic acid-binding immunoglobulin-like lectin
SUV: Standardized uptake value
VAP-1: Vascular adhesion protein-1
